# Inflammation of the Pulps of Ten Teeth

**Published:** 1853-04

**Authors:** John Bilisurio

**Affiliations:** Dentist. Sydney, Australia.


### ARTICLE IX.

*Inflammation of the Pulps of Ten Teeth.* By JOHN BILISURIO, Dentist. Sydney, Australia.

A gentleman, aged twenty-five years, on his passage from England to this colony: when off the cape, he got a severe wetting, and a few hours afterwards, was attacked with a dull aching pain all along the base of the lower jaw, which pain lasted about twenty-four hours, increasing in intensity all the time, and was then succeeded by throbbing—to use the patient's own words—the agony was so great as to be scarcely endurable.

The face slightly swelled. At the expiration of thirty-six hours, the pain gradually decreased—after the attack he found great difficulty in opening the mouth to any extent—mastication performed with much pain—swelling still continued the same, and a fetid taste always in the mouth. About this time, he says, his general health was much impaired. On his arrival at Sydney he consulted an eminent medical man, when after taking various constitutional remedies without producing any effect, he requested me to see him, and the following were the appearances presented: On examination of his mouth, which could hardly be opened to the extent of a quarter of an inch without producing very great pain; all the upper teeth were perfectly free from disease; the six lower molars had been removed, the remaining ten had a slightly bluish appearance, and immediately opposite the root of each tooth had been an abscess with a consequent opening for matter. In fact the whole of the outer plate of bone was nearly destroyed, and a large quantity of matter was consequently exuding from the openings which at once accounted for the irritation and pain the patient suffered.

I have seen many cases of general inflammation of the pulp ending in suppuration and alveolar abscess from one or two teeth, but I have never seen, heard or read of a case of so many teeth becoming similarly affected at once. After a careful consideration of the case the only plan of treatment which I could adopt, and which was concurred in by his medical adviser, was the removal of the remaining ten teeth, which my patient at once consented to, providing he was chloroformed. The roots of the teeth presented the usual appearances resulting from abscess. Leeches were applied to the angles of the jaws, followed by aperients and tonics. It is now four months since the operation was performed. The mouth is perfectly healthy and can be opened to the full extent. The general health is good. I have since supplied the defect artificially.

